# Supplementary material for: Patterns of Intron Gain and Loss in Fungi
Source: PLoS Biol. 2004 Nov 30;2(12):e422. doi: 10.1371/journal.pbio.0020422 (PMC532390; doi:10.1371/journal.pbio.0020422)
Supplement: Table S1 — Also available at http://genes.mit.edu/NielsenEtAl/. (4.3 MB ZIP). [file pbio.0020422.st001.zip › NielsenEtAl/html/447.html]

AN3411.1.NCU02076.1.MG04647.1.FG00286.1


```
 CLUSTAL W (1.82) Multiple Sequence Alignments - Introns Inserted


Sequence 1: NCU02076.1	248 aa
Sequence 2: MG04647.1	244 aa
Sequence 3: FG00286.1	190 aa
Sequence 4: AN3411.1	244 aa
Alignment Length: 252 aa
Number Identitical Residues: 117 aa
Alignment Score (without introns) 6302


MG04647.1 	MSTQVDLSTIPISPDGEN----APTSSGDKE----VTVFHDSNNFNVKHPLMHKWTLWFT
NCU02076.1	MSEQVDLTTIPISPEGGNTNGDAAAAKDDKT----VTVFHDKEHFNVKHPLSNRWTLWFT
FG00286.1 	------------------------------------------------------------
AN3411.1  	-MAAVAENAPPVTNENTAPPAVAVEEKTEKANGDIVTVFHDPENFNVKHPLMHEWTLWFT
          	       .: . : ..  . . :   . .. ...  :   . .  . . .     :   :

MG04647.1 	KPPTGK0GDNWAELLKEVITFNSVEEFWGIY0NNIAPVSELALKSDYHLFKEGVRPEWED
NCU02076.1	KPSSGK0GDNWNDLLKEVITFETVEEFWGVY0NNIAPVSELALKSDYHLFKEGVRPEWED
FG00286.1 	--MDEQ0GDNWNDLLKEVITFDSVEEFWGVY0NNVAPVSELSLKSDYHLFKEGVRPEWED
AN3411.1  	KPPSGK0GDNWNDLLKEVVTFNSVEEFWGIY0NNITPTSELGLKADYHLFKKGVRPEWED
          	..   : **** :*****:**::******:* **::*.***.**:******:********

MG04647.1 	PQNKHGGKWSYSFKEKRNIPIDDIWLHTMLAAIGETLESEDDGEVMGVVVNVRKAFYRVG
NCU02076.1	PQNKHGGKWSYQFKEKRNVNIDEIWLHTMLAAIGETLEDEEDGEVMGVVVNVRKAFFRVG
FG00286.1 	PQNKHGGKWSYQYKDKRNIDIDRLWLQVMMGAIGETLENEDDGEVMGVVVNVRKAFFRIG
AN3411.1  	PQNKHGGKWSFSFKDKRSVPIDDLWLHAQLAAIGETLENDGDNEVMGVVVNVRKGFYRVG
          	**********:.:*:**.: ** :**:. :.*******.: *.***********.*:*:*

MG04647.1 	VWTRTTGKSIPNRGDGDVAGGKGRSPEKGRAILMDIGKRFKDVLRLPANEVLEFSGHTES
NCU02076.1	VWTRTIGKHIPGRGDGDISGGKGRSPEKGKEILMAIGRRFKEVLQLPPNEALEFSGHTES
FG00286.1 	VWTRTIGKSIPGRGEGDVAGGKGRSGEKGKEILLSIGRRFKEVLELPASEQVEFSGHTDS
AN3411.1  	LWTRTVGKTLP----GDKSG---RTPAQGKEVLESIGRRFKEVLRLGPNDSVEFSGHTDS
          	:**** ** :*    ** :*   *:  :*: :*  **:***:**.* ..: :******:*

MG04647.1 	AHSGSTRAKAKFTV
NCU02076.1	AHSGSSRARAKMTV
FG00286.1 	AHSGSTRAKAKHVV
AN3411.1  	AHSGSTRAKAKYTV
          	*****:**:** .*
```
